# Transcriptome profiling revealed potentially important roles of defensive gene expression in the divergence of insect biotypes: a case study with the cereal aphid *Sitobion avenae*

**DOI:** 10.1186/s12864-020-06950-y

**Published:** 2020-08-06

**Authors:** Da Wang, Deguang Liu, Xiaoqin Shi, Yujing Yang, Na Zhang, Zheming Shang

**Affiliations:** 1 State Key Laboratory of Crop Stress Biology for Arid Areas (Northwest A&F University), Yangling, 712100 Shaanxi China; 2 College of Plant Protection, Northwest A&F University, Yangling, 712100 Shaanxi China

**Keywords:** Grain aphid, Biotype differentiation, Population divergence, Phenotypic plasticity, RNA-seq, Adaptive evolution

## Abstract

**Background:**

Many insects can develop differential biotypes on variable host plants, but the underlying molecular factors and mechanisms are not well understood. To address this issue, transcriptome profiling analyses were conducted for two biotypes of the cereal aphid, *Sitobion avenae* (Fabricius), on both original and alternative plants.

**Results:**

Comparisons between both biotypes generated 4174 differentially expressed unigenes (DEGs). In their response to host plant shift, 39 DEGs were shared by both biotypes, whereas 126 and 861 DEGs occurred only in biotypes 1 and 3, respectively. MMC (modulated modularity clustering) analyses showed that specific DEGs of biotypes 1 and 3 clustered into five and nine transcriptional modules, respectively. Among these DEGs, defense-related genes underwent intensive expression restructuring in both biotypes. However, biotype 3 was found to have relatively lower gene transcriptional plasticity than biotype 1. Gene enrichment analyses of the abovementioned modules showed functional divergence in defensive DEGs for the two biotypes in response to host transfer. The expression plasticity for some defense related genes was showed to be directly related to fecundity of *S. avenae* biotypes on both original and alternative plants, suggesting that expression plasticity of key defensive genes could have significant impacts on the adaptive potential and differentiation of *S. avenae* biotypes on different plants.

**Conclusions:**

The divergence patterns of transcriptional plasticity in defense related genes may play important roles in the phenotypic evolution and differentiation of *S. avenae* biotypes. Our results can provide insights into the role of gene expression plasticity in the divergence of insect biotypes and adaptive evolution of insect populations.

## Background

More than 90% of phytophagous insects only feed on one or a few host plant families [1, 2]. Thus, many insect populations often evolve host plant-specific adaptations, forming different biotypes [3]. Following Painter [4] and Smith [5], biotypes in this study are defined as populations within an insect species that display unique response profiles on a set of resistant host plants (i.e., different plant species or different varieties of the same plant). Biotype development in insects provides excellent models for understanding local adaptation and genetic changes, and has long been a focus of evolutionary and ecological research [5–9]. About 50% of all the insects with known biotypes belong to the family Aphididae [10]. The divergence of biotypes has been found to occur in many aphids like the pea aphid (*Acyrthosiphon pisum*), Russian wheat aphid (*Diuraphis noxia*), greenbug (*Schizaphis graminum*) and the soybean aphid (*Aphis glycines*) [11–19]. One possible explanation for this phenomenon is that significant genetic divergence often occur in different geographic populations or host-associated clones for aphids, which can be important in promoting the development of biotypes [20–23]. Phenotypic plasticity seems to be a particularly common phenomenon for different populations of aphids compared with other insect groups [24–27]. Thus, another possible explanation is that phenotypic plasticity and underlying gene expression plasticity may make aphids highly amenable to development of variable biotypes, but direct evidence is rare.

Indeed, some researches have strongly suggested that selective expression of genes can play an important role in regulating plastic phenotypes [28–31]. Additionally, it is believed that environmentally induced shifts in gene expression are plasticity operating at the most fundamental level [32, 33]. Recent advances in genomic technologies have made it possible to detect the transcriptional plasticity of organisms responding to variable environments [34, 35]. Gene expression profiles can provide more phenotypes that can easily be documented [35]. Using a whole-transcriptome sequencing, the gene expression plasticity was detected to be differed between freshwater and marine three-spine stickleback (*Gasterosteus aculeatus*) ecotypes in response to temperature and salinity acclimation, which indicated that the plastic expression of genes could play an important role in colonization and adaptation to new environments [36, 37]. Transcriptional profiling can also allow the simultaneous assessment of the magnitude for transcriptional plastic responses to environmental shifts, as well as the biological functions involved [38, 39]. Thus, this can be a valuable approach for exploring fundamental changes underlying the divergence of biotypes in insects, for which the molecular factors involved and underlying mechanisms are little understood [9, 35, 39, 40].

The English grain aphid, *Sitobion avenae* (Fabricius), provides a good model to address these issues. It is a significant worldwide pest on cereal crops, and it can survive on many species of cereals and grasses [41–44]. Some studies have found certain degrees of plant specialization in *S. avenae* [23, 45–47]. In our previous study, based on their unique virulence profiles on different resistant cultivars of wheat and barley, multiple biotypes of *S. avenae* were identified [48]. However, only little to moderate genetic differentiation was detected among *S. avenae* biotypes, which could not explain the divergence of biotypes in this aphid [48]. In this study, we have examined gene expression of two biotypes (i.e., biotypes 1 and 3) on both original and alternative hosts by deeply sequencing the entire transcriptome. The specific objectives are to: 1) examine differential gene expression in both *S. avenae* biotypes in response to host plant shift; and 2) explore molecular factors and mechanisms underlying the divergence of *S. avenae* biotypes.

## Results

### Transcriptome assembly and annotation

A total of 337,188,919 clean reads were generated from two aphid biotypes on wheat and barley. Over 78.62, 82.17, 80.98 and 95.42 million clean reads were respectively found in transcriptome sequencing of aphid samples in the four treatment: AW (biotype 1 on wheat), AB (biotype 1 on barley), BW (biotype 3 feeds on wheat), and BB (biotype 3 feeds on barley) (Table S1). Each sample library was mapped back to the full assembly with an overall alignment rate of 71.67–73.23%. Using the combined dataset, de novo assembly for *S. avenae*’s transcriptome produced 143,058 unigenes (a total of 95,512,520 bases) with a mean length of 358 nt. The N50 (length N for which 50% of all bases in the assembly are located in a transcript of length L < N) of the assembly equaled 1012. The BUSCO analysis showed a level of 94.9% completeness for the assembly (67.2% complete and single-copy orthologs and 27.7% complete and duplicated orthologs) (Fig. S1), showing the quality of the assembly and annotation completeness. The results of the principal component analysis (PCA) showed that the four treatments were clearly separated in the plot, and three biological replicates for each treatment clustered together, indicating all biological replications of each treatment had good repeatability (Fig. S2).

### Transcriptional plasticity at the transcriptome level

**Fig. 1:**
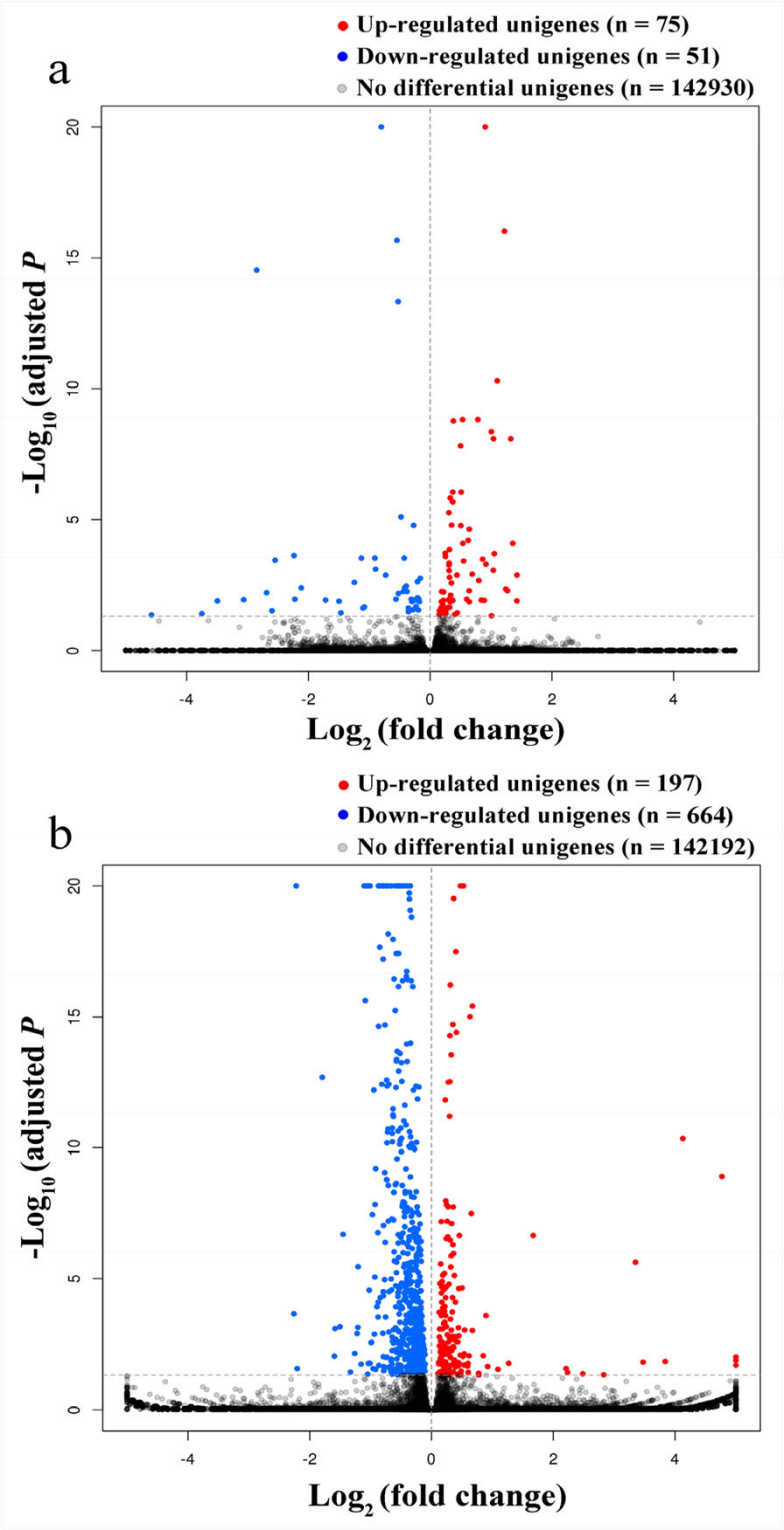
Gene expression changes in biotypes 1 (**a**) and 3 (**b**) responding to host transfer (gene expression levels between treatments were considered significantly different if adjusted *P* < 0.05)

**Fig. 2:**
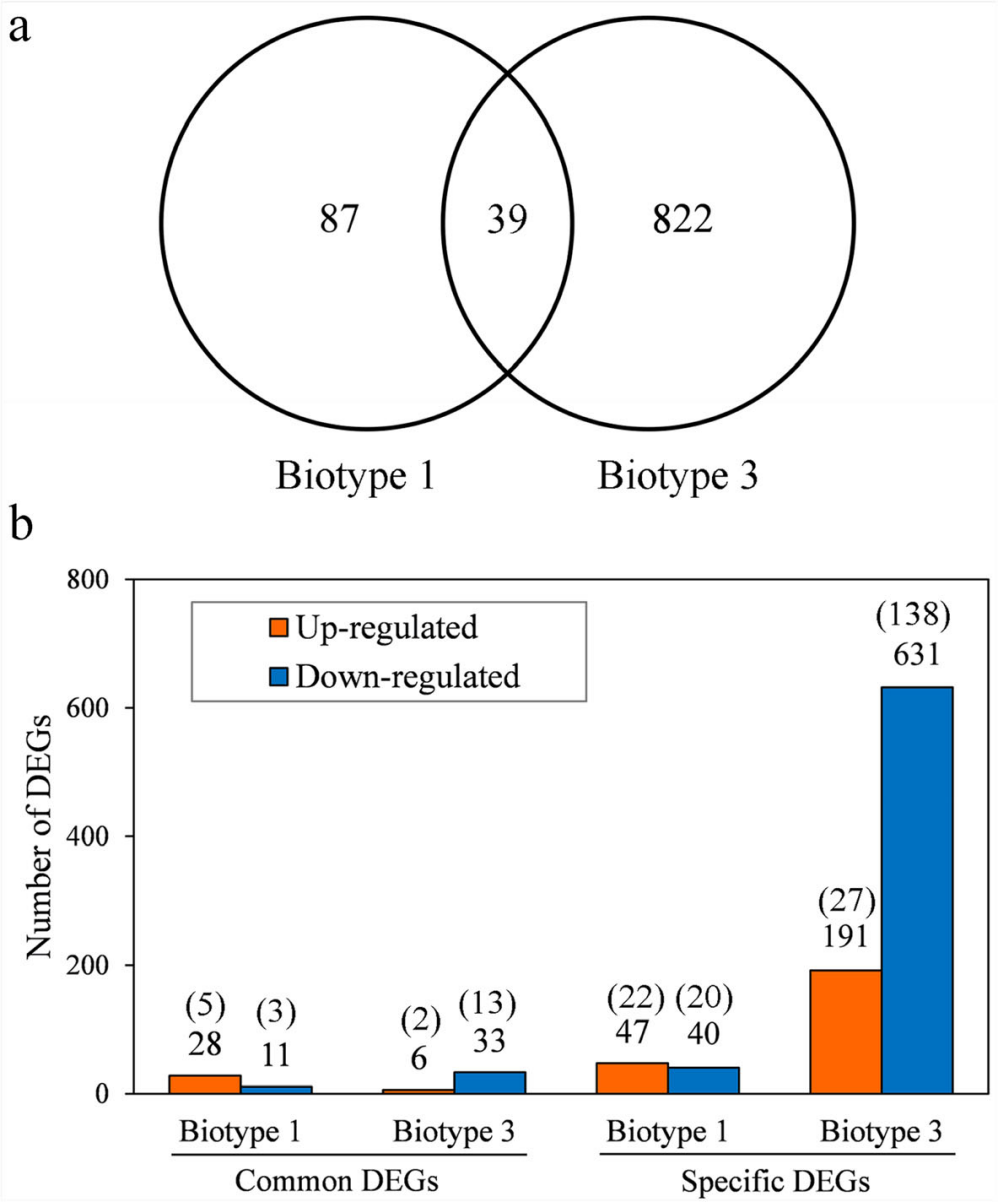
Comparisons on the numbers of common and specific DEGs in *Sitobion avenae* biotypes 1 and 3 in response to host plant transfer (DEGs, differentially expressed genes; **a** the total number of DEGs; **b** counts of up- and down-regulated DEGs; DEGs co-existed in the two aphid biotypes were considered to be common DEGs, otherwise, they were considered specific DEGs; adjusted *P* < 0.05; the numbers of DEGs with fold change > 1.5 were shown in brackets above bars)

Of all the 143,058 transcripts, 4174 differentially expressed unigenes (DEGs) was detected between two *S. avenae* biotypes (adjusted *P* value < 0.05) (Table 1). GO-enrichment analysis (FDR < 0.005) of these DEGs demonstrated enrichment of multiple terms of biological processes associated with aphid defense (e.g., response to toxic substance, oxidation-reduction process, detoxification, proteolysis, chitin metabolic process, and etc.) (Table 1). To characterize transcriptome plasticity, we identified DEGs for each biotype responding to host plant transfer, and 126 (75 upregulated, 51 downregulated) and 861 (197 upregulated, 664 downregulated) unigenes were differentially expressed (adjusted *P* value < 0.05) after host plant transfer in biotypes 1 and 3, respectively (Fig. 1). Of these DEGs, we identified many transcripts related to detoxification and defense in aphids (Table 2), including those encoding for cytochrome P450s (2 in biotype 1 and 8 in biotype 3), carboxylesterase (0 in biotype 1 and 2 in biotype 3), UDP-glucuronosyltransferases (2 in biotype 1 and 6 in biotype 3, 1 co-existed in two biotypes), protease inhibitor, peroxidase, heat shock protein, and etc. Thirty nine DEGs occurred in both two biotypes (Fig. 2). Eighty seven (47 upregulated, 40 downregulated) and 822 (191 upregulated, 631 downregulated) DEGs occurred only in biotypes 1 and 3, respectively.

**Table 1:** Top 20 enriched biological process GO-terms for genes that were differentially expressed between *Sitobion avenae* biotypes 1 and 3

GO	Go_description	DEGs	Total	*P*-value	FDR
GO:0044699	Single-organism process	851	7246	< 0.001	0.003
GO:0065007	Biological regulation	360	2643	0.001	< 0.001
GO:0050789	Regulation of biological process	339	2495	< 0.001	< 0.001
GO:0050794	Regulation of cellular process	321	2385	< 0.001	< 0.001
GO:1902578	Single-organism localization	247	1771	< 0.001	< 0.001
GO:0044765	Single-organism transport	245	1753	< 0.001	< 0.001
GO:0055114	Oxidation-reduction process	237	1680	< 0.001	< 0.001
GO:0055085	Transmembrane transport	183	1220	< 0.001	< 0.001
GO:0006508	Proteolysis	172	987	< 0.001	< 0.001
GO:0006629	Lipid metabolic process	73	382	< 0.001	< 0.001
GO:0008610	Lipid biosynthetic process	45	192	< 0.001	< 0.001
GO:0009636	Response to toxic substance	34	137	< 0.001	< 0.001
GO:0098754	Detoxification	33	136	< 0.001	< 0.001
GO:0098869	Cellular oxidant detoxification	33	136	0.001	< 0.001
GO:1990748	Cellular detoxification	33	136	< 0.001	< 0.001
GO:1901071	Glucosamine-containing compound metabolic process	26	92	< 0.001	< 0.001
GO:0006040	Amino sugar metabolic process	26	101	0.001	0.002
GO:0006030	Chitin metabolic process	25	82	< 0.001	< 0.001
GO:0072330	Monocarboxylic acid biosynthetic process	23	67	< 0.001	< 0.001
GO:0006633	Fatty acid biosynthetic process	21	62	< 0.001	< 0.001

The total number of differentially expressed genes between the two biotypes on the original plant was 4174; all terms significantly enriched with FDR < 0.005

*DEGs* differentially expressed genes

**Table 2:** Defense related DEGs (differentially expressed genes) in *Sitobion avenae* biotypes 1 and 3 in response to host plant transfer

Genes	^a^present	^b^Common DEGs	^c^Specific DEGs	
			Biotype 1	Biotype 3
Cytochrome P450	122	0	2	8
Carboxylesterase	16	0	0	2
Glutathione S-transferase	73	1	0	1
UDP-glucuronosyltransferase	97	1	1	5
Esterase	31	0	0	4
ABC transporter	214	0	0	3
Alkaline phosphatase	14	1	0	0
Peroxidase	109	0	0	5
Superoxide dismutase	20	0	0	1
Flavin-containing monooxygenase	1	1	0	0
Laccase	10	0	1	2
Protease inhibitor	13	0	0	1
Cuticle protein	63	1	2	15
Zinc transporter	29	0	1	2
Heat shock protein	104	0	1	6
Serine protease	107	0	1	2
Cysteine protease	28	3	1	0
Trehalose phosphate synthase	14	1	0	1

^a^the total number of transcripts identified

^b^the number of differentially expressed genes co-existed in the two biotypes

^c^the number of differentially expressed genes occurred only in one biotype

**Fig. 3:**
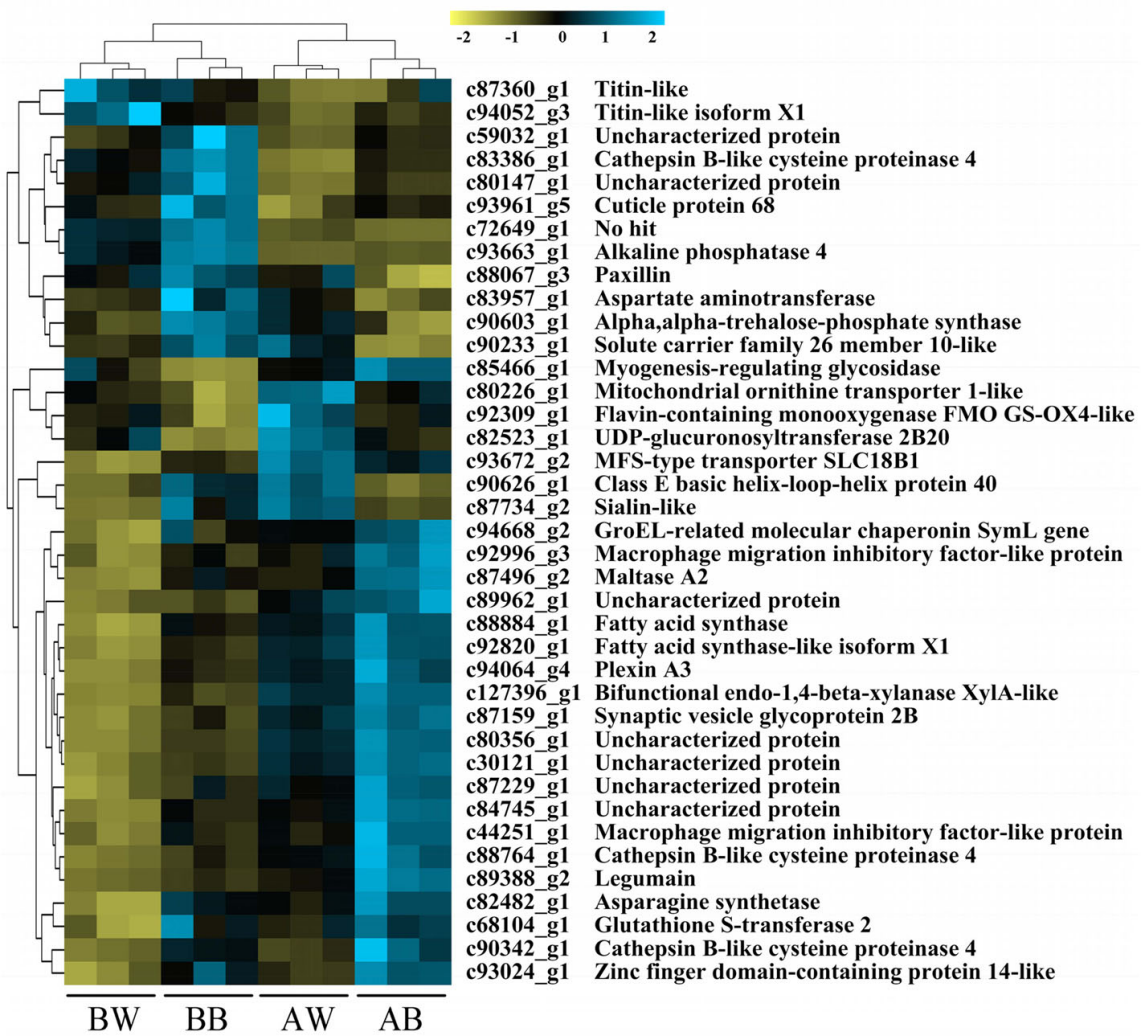
A heatmap displaying expression patterns of common DEGs in two *Sitobion avenae* biotypes in response to host plant transfer (blue, genes with expression higher than the mean; yellow, genes with expression lower than the mean; BW, biotype 3 feeds on wheat; BB, biotype 3 feeds on barley; AW, biotype 1 feeds on wheat; AB, biotype 1 feeds on barley)

Among the 39 common DEGs, eight were predicted with unknown function, and 31 were annotated (Fig. 3). In response to host plant transfer, 11 DEGs had consistent expression change pattern (up-regulated or down-regulated) for both biotypes. However, the expression pattern of the other 28 DEGs for biotype 1 was in contrast to that of biotype 3. Of the common DEGs related to xenobiotic metabolism and defense of *S. avenae*, the UDP-glucuronosyltransferase gene (*UGT2B20*) was down-regulated in biotype 1, but up-regulated in biotype 3, and the same pattern was found for the flavin-containing monooxygenase gene (*FMO GS-OX4*). The glutathione S-transferase gene (*GST2*) was up-regulated in biotype 1, but down-regulated in biotype 3, and the same pattern was detected for the alkaline phosphatase gene (*ALP4*), the three Cathepsin B-like cysteine proteinase genes (i.e., *CBCP4–1*, *CBCP4–2*, *CBCP4–3*), and the cuticle protein gene (*CP68*). The alpha-trehalose-phosphate synthase gene (*TPS*) were up-regulated for both biotypes.

Ten biotype 1-specific DEGs related to aphid defense or stress response were detected, including two cytochrome P450s, one UDP-glycosyltransferase, one laccase, one zinc transporter, one HSP70 protein, one serine protease, one cysteine proteinase and two cuticle proteins (Table S2). All of them were up-regulated except two cytochrome P450s and one UDP-glycosyltransferase. Fifty-eight biotype 3-specific DEGs were found to be associated with defense or stress response, including eight P450s, two carboxylesterases, four esterases, one glutathione S-transferase, five UDP-glucuronosyltransferases, three ABC transporters, one cytochrome b5, five peroxidases, one superoxide dismutase, two laccases, one protease inhibitor, 14 cuticle proteins, two zinc transporters, six heat shock proteins, two serine protease and one trehalose phosphate synthase (Table S3). Of them, two P450s, one UDP-glucuronosyltransferase, the protease inhibitor, one zinc transporter, and one heat shock protein were upregulated, and the other 52 DEGs were downregulated.

**Fig. 4:**
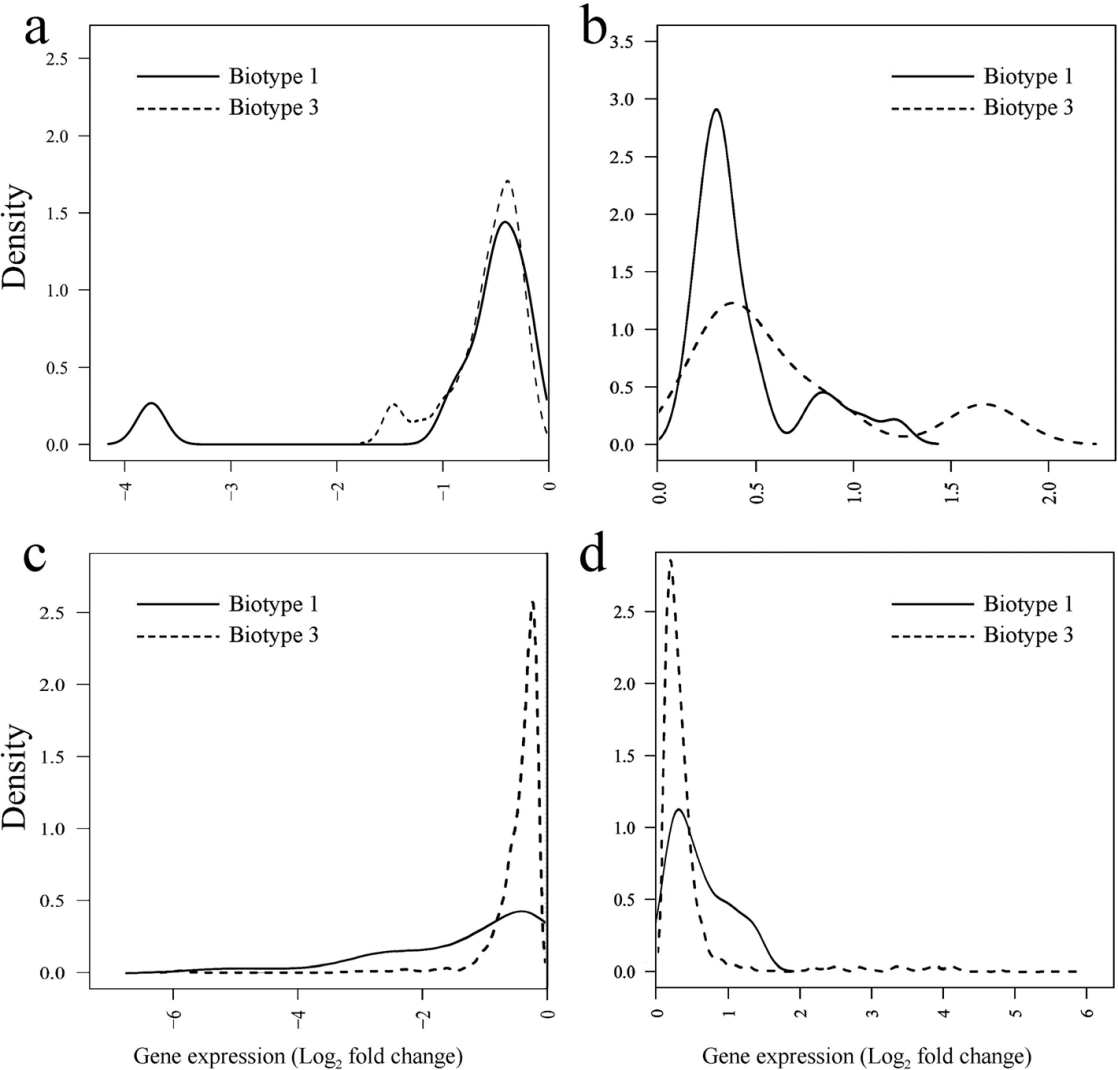
Differences between *Sitobion avenae* biotypes 1 and 3 in the distribution of Log_2_ fold changes of common DEGs (**a** and **b**, for genes downregulated and upregulated, respectively) and specific DEGs (c and d, for genes downregulated and upregulated, respectively) in response to host plant transfer [Lines represent the relative density (amount) of genes corresponding to the fold changes indicated on the x-axis for biotype 1 (solid lines) and biotype 3 (dashed lines); the relatively higher density of genes at a low range of magnitudes of Log_2_ fold change indicates a reduced scope for transcriptional plasticity; the shift of the distribution between biotypes is statistically significant for comparisons in both (**c**) and (**d**) based on the Wilcoxon rank-sum test]

The magnitudes of Log2 fold changes of all DEGs in the two biotypes in response to host transfer were compared, and the distributions of Log2 fold changes of DEGs were created to show the scope for transcriptional plasticity in the two biotypes. We found that the mean magnitude of Log2 fold change for specific DEGs (upregulated and downregulated) of biotype 1 was higher than that of biotype 3 (*P* < 0.001, Table 3). At low ranges of Log2 fold changes, the density of DEGs of biotype 3 tended to be higher than that of biotype 1 (Fig. 4c and d), indicating a relatively lower scope of transcriptional plasticity for both upregulated and downregulated specific DEGs in biotype 3. There were no significant differences between the two biotypes for the magnitude of Log_2_ fold changes for upregulated and downregulated common DEGs (*P* = 0.125 and 0.769, Table 3). Similar density distributions of common DEGs were found between the two biotypes (Fig. 4a and b).

**Table 3:** Magnitude of Log_2_ fold changes for DEGs in *Sitobion avenae* biotypes 1 and 3 in response to host plant transfer

		Biotype 1		Biotype 3		Wilcoxon W	*p* value
		N	Mean (SE)	N	Mean (SE)		
Common DEGs	Upregulated	28	0.43 (0.052)	6	0.67 (0.218)	456	0.125
	Downregulated	11	0.76 (0.306)	33	0.58 (0.059)	731	0.769
Specific DEGs	Upregulated	47	0.61 (0.058)	191	0.53 (0.068)	20,984	< 0.001
	Downregulated	40	1.14 (0.178)	631	0.40 (0.011)	8629	< 0.001

Differentially expressed genes (DEGs) occurred in one biotype only in response to host plant transfer were considered specific

*N* number of unigenes, *Wilcoxon W* W statistic for the Wilcoxon rank-sum test

### MMC and correlational analysis

**Fig. 5:**
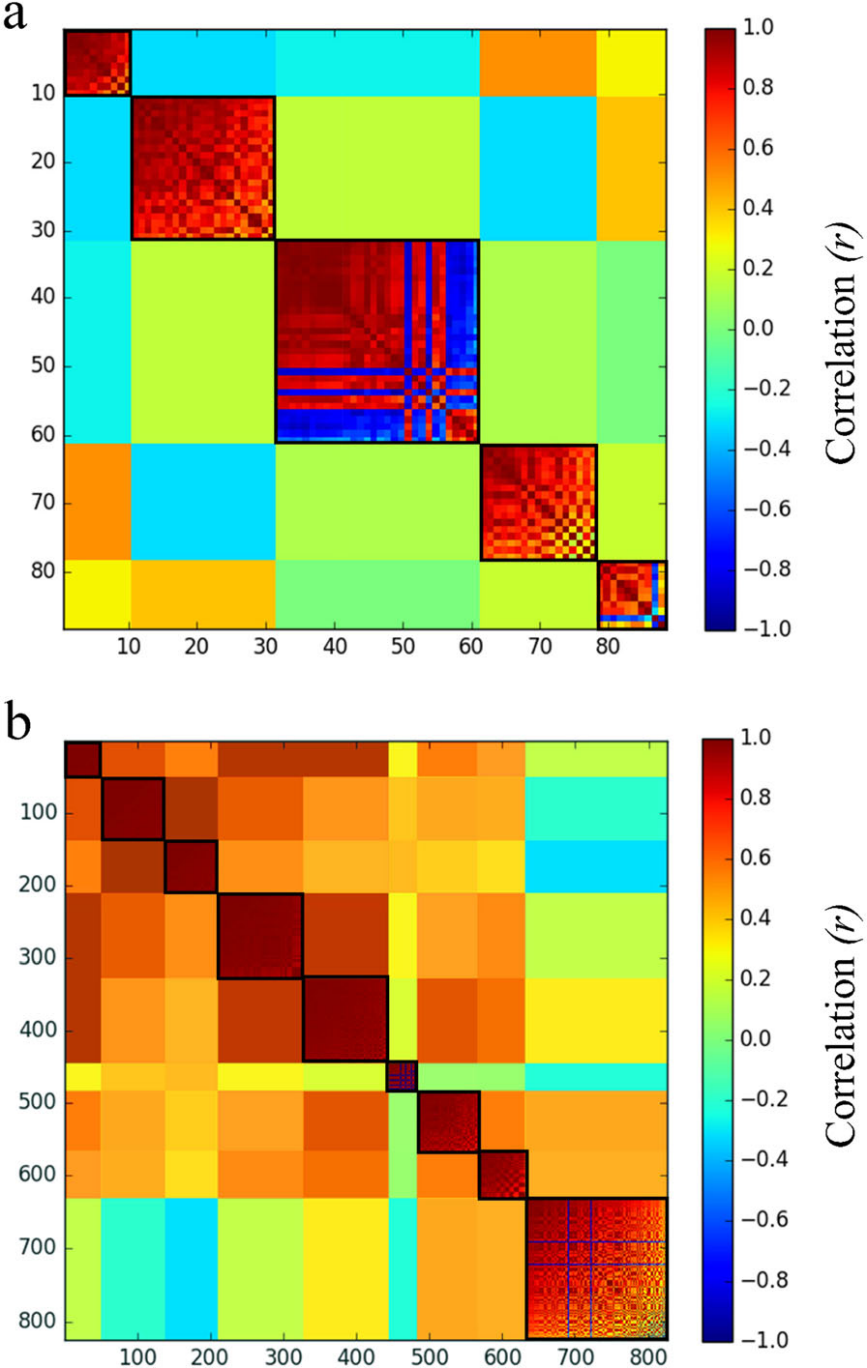
Transcriptional modules obtained by the modulated modularity clustering analysis of specific DEGs in biotypes 1 (**a** 87 transcripts fell into five modules) and 3 (**b** 822 transcripts fell into nine modules) in response to host plant transfer

For specific DEGs of each biotype in response to host plant transfer, we calculated correlation coefficients for all pairwise gene expression values, and then used MMC analysis to identify modules of highly inter-correlated and co-regulated genes. This analysis generated five modules (i.e., P1-P5) from 87 specific DEGs of biotype 1 (Fig. 5a), and nine modules (i.e., T1-T9) from 822 specific DEGs of biotype 3 (Fig. 5b). The large number of modules and their small sizes reflect the overall heterogeneity in transcriptional plasticity. The heterogeneity of plastic transcription modules appeared to be more pronounced in biotype 3 than in biotype 1. The modules of specific DEGs of biotype 1 were significantly enriched for gene ontology (GO) terms related to drug metabolic process (P1) and proteolysis (P2, Table S4). For specific DEGs of biotype 3, GO terms were significantly enriched for protein folding in the module T1, for peptide metabolic process in the module T2, for chitin metabolic process and regulation of protein metabolic process in the module T4, for oxidation-reduction process in the module T7, for transmembrane transport in the module T8, and for transmembrane transport in the module T9 (Table S4). Go enrichment analyses clearly indicated that there was functional divergence between DEGs of the two biotypes in response to host transfer.

For validation purposes, we selected 14 representative genes (one defensive gene each module) from all transcriptional modules identified above, and examined their expression levels in both biotypes in response to host plant transfer by using qRT-PCR. Except for the cytochrome b5 reductase 2 gene (*CYB5R2*), all the selected DEGs were significantly upregulated or downregulated in both biotypes (Fig. S3a). In addition, a significant correlation (*r* = 0.954; *P* < 0.001) was found between data sets of RNA-Seq and qRT-PCR, showing consistency of both analyses (Fig. S3b).

**Fig. 6:**
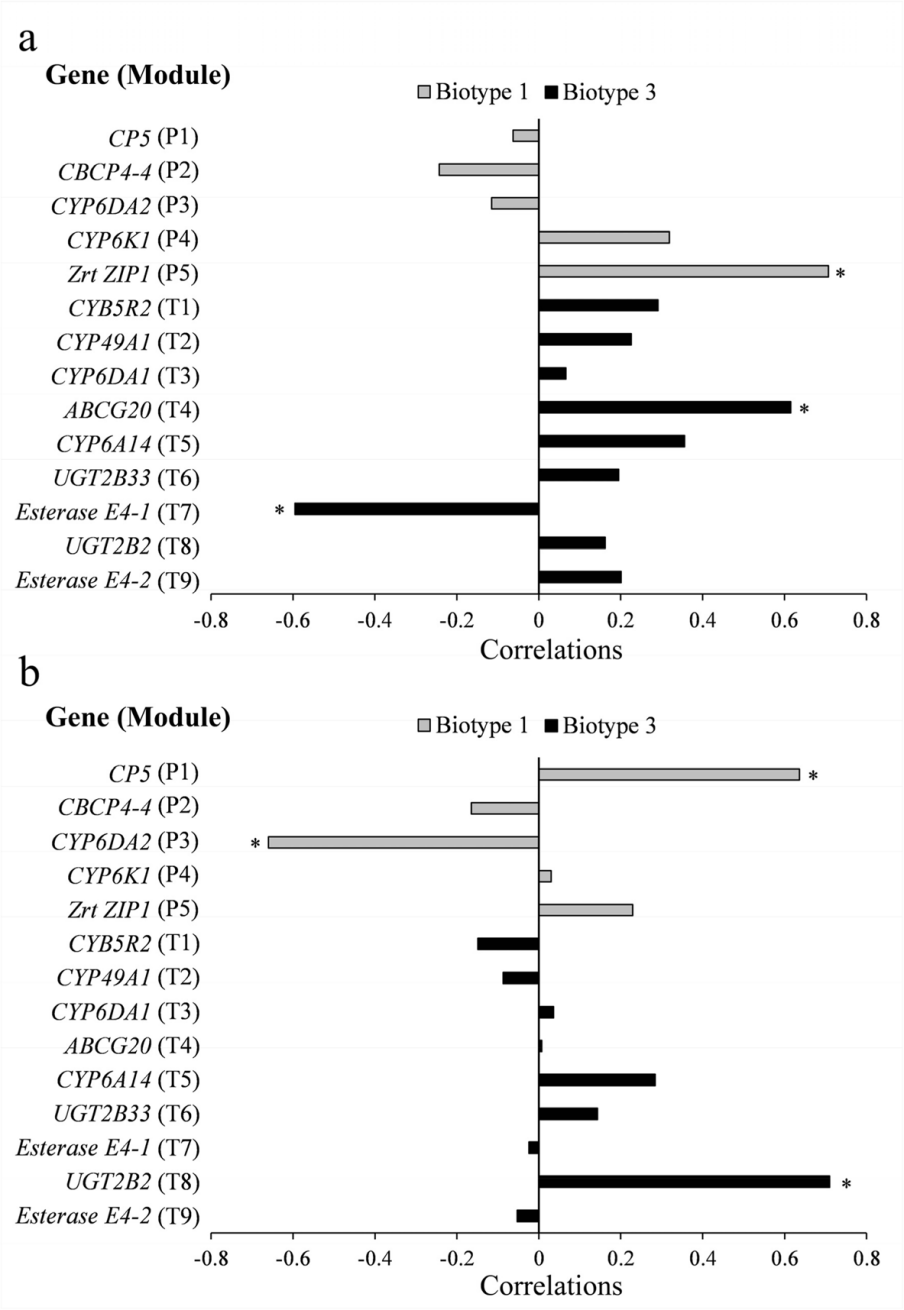
Pearson correlations between five-day fecundity and the expression of a representative gene in each transcriptional module for both biotypes of *Sitobion avenae* on the original (**a**) or alternative plant (**b**) (*CP5*, cuticle protein 5-like, c63100_g1; *CBCP4–4*, cathepsin B-like cysteine proteinase 4, c90342_g4; *CYP6DA2*, cytochrome P450 6DA2, c77510_g1; *CYP6K1*, cytochrome P450 6 k1-like, c92410_g2; *Zrt ZIP1*, zinc transporter ZIP1, c89079_g2; *CYB5R2*, cytochrome b5 reductase 2, c85139_g1; *CYP49A1*, cytochrome P450 49a1, c80471_g1; *CYP6DA1*, cytochrome P450 6DA1, c82766_g1; *ABCG20*, ABC transporter G family member 20, c94097_g2; *CYP6A14*, cytochrome P450 6a14, c94331_g1; *UGT2B33*, UDP-glucuronosyltransferase 2B33, c90721_g1; *Esterase E4–1*, Esterase E4-like, c94328_g3; *UGT2B2*, UDP-glucuronosyltransferase 2B2, c92512_g4; *Esterase E4–2*, Esterase E4,c88302_g1; *, *P* < 0.05)

We further analyzed the correlations between expression levels of representative genes in identified transcriptional modules and five-day fecundities (i.e., a fitness surrogate) of each biotype on both wheat and barley. The expression of the zinc transporter ZIP1 gene (*Zrt ZIP1*) in the P5 module was significantly correlated with the fecundity of biotype 1 on its original plant (i.e., wheat) (Fig. 6a; *r* = 0.707, *P* = 0.001). The fecundity of this biotype on its alternative plant (i.e., barley) had a significantly positive correlation with the expression of the cuticle protein gene (*CP5*) in the P1 module (Fig. 6b; *r* = 0.636, *P* = 0.026), but a significantly negative correlation with the expression of *CYP6DA2* (a cytochrome P450 gene) in the P3 module (*r* = − 0.660, *P* = 0.020), showing potentially critical roles of DEGs in these modules for biotype 1 on a resistant alternative plant. The fecundity of biotype 3 on the original plant (i.e., barley) correlated with the expression of *ABCG20* (ABC transporter G family member) in the T4 module and the esterase E4-like gene *Esterase E4–1* in the T7 module. In addition, the fecundity of biotype 3 on the alternative plant (i.e., wheat) was significantly correlated with the expression of the UDP-glucuronosyltransferase gene *UGT2B2* in the T8 module (*r* = 0.710, *P* = 0.010), showing the significance of this module of DEGs for biotype 3 on a resistant alternative plant.

## Discussion

Many insect species, esp. aphids, can survive and develop into differential biotypes (or host races) on variable host plants, but the interactions between these insects and their respective host plants are not well understood. Research on molecular aspects of these interactions may provide insights into molecular and genetic mechanisms underlying development and evolution of insect biotypes. The English grain aphid (*Sitobion avenae*) provides a good model to address these issues. This aphid can survive on many cereals and wild grasses in the Poaceae, and has been found to be able to evolve multiple biotypes on both barley and wheat [48]. Among all the *S. avenae* biotypes, biotype 3 was found to have relatively higher fitness parameters on resistant barley varieties (e.g., cv. Xiyin No.2) than on wheat varieties (e.g., Aikang 58) [48]. The opposite was true for biotype 1, indicating clearly that adaptive differentiation had occurred between the two biotypes. Of all the 143,058 transcripts, 4174 differentially expressed unigenes (DEGs) was detected between two *S. avenae* biotypes. The enriched GO-terms of these DEGs demonstrated that there had been expression divergence in genes associated with defense (e.g., detoxification, response to toxic substance, oxidation-reduction process, proteolysis, and chitin metabolic process) for the two biotypes. The gene expression responses to host plant shift at the transcriptome level were also compared for the two *S. avenae* biotypes. We found that, in response to host plant transfer, 126 and 861 unigenes were differentially expressed in *S. avenae* biotypes 1 and 3, respectively. Of all the above-mentioned DEGs in *S. avenae*’s response to host plant transfer, 39 were shared by both biotypes tested. These common DEGs encoded for one UDP-glucuronosyltransferase (*UGT2B20*), one glutathione S-transferase (*GST2*), one flavin-containing monooxygenase (*FMO GS-OX4*), one alkaline phosphatase (*ALP4*), three cysteine proteases (*CBCP4–1*, *CBCP4–2*, *CBCP4–3*), and one cuticle protein (*CP68*), and one trehalose synthase (*TPS*). In addition to UGT and GST (two major classes of phase II detoxification enzymes in insects), FMO in insects can catalyze the conversion of heteroatom-containing xenobiotics to polar, readily excretable metabolites [49–51]. ALP is generally considered to be a hydrolase involved in insect resistance to pesticides and xenobiotics [52–55]. The expressional response of cysteine proteases in this aphid can be attributed to protease inhibitors present in both wheat and barley [56, 57]. Cuticular proteins, essential in gut membrane recombination, can restrict the movement of toxicants from gut to haemocoel [58, 59]. As one of the most important genes involved in the trehalose synthesis process, TPS has been extensively studied in insect stress resistance [60, 61]. Thus, most of these common DEGs were found to be associated with detoxification and defense of *S. avenae*, which can be very important in the evolution of various biotypes in different aphid populations. Further functional studies of such common DEGs among aphid biotypes may reveal their critical roles in the evolution of various biotypes in different aphid populations.

In addition to common DEGs, a large number of biotype-specific DEGs (126 in biotype 1 and 861 in biotype 3) were also identified. It has been suggested that specific expression of certain genes in different aphid populations may also play a key role in the divergence of aphid biotypes [9, 40, 62]. Indeed, our MMC and correlation analyses with biotype-specific DEGs showed that the fecundity of biotype 1 was significantly correlated with the expression of the cuticle protein gene (*CP5*) in the module P1 and the cytochrome P450 gene (*CYP6DA2*) in the module P3, suggesting both transcriptional modules had significant functional implications for colonization of alternative plants by biotype 1. Similarly, the fecundity of biotype 3 showed strong associations with expression of the UDP-glucuronosyltransferase gene (*UGT2B2*) in the module T8. GO enrichment analyses showed that GO terms of transcription modules P1 and P2 for biotype 1-specific DEGs were significantly enriched in “drug metabolic process” and “proteolysis”, respectively. “Drug metabolism” can play central roles in the detoxification of xenobiotics introduced into the body of various organisms including aphids [63, 64]. In addition to providing supplementary supply of organic N-compounds to the aphid diet, proteolysis can also be important in the sabotage of protein-mediated plant defense mechanisms [65]. Among biotype 3-specific DEGs, genes in the transcriptional module T7 were enriched for functions of “oxidation-reduction process”. For insects, monooxygenase (e.g., P450s) and antioxidant (e.g., superoxide dismutase, catalase, glutathione transferase, and glutathione reductase) systems are frequently involved in this process, and have functions of detoxification and protection [66, 67]. The biological processes of “chitin metabolic process” and “regulation of protein metabolic process” were significantly enriched in the module T4. Chitin and protein metabolisms not only are critical to the development and reproduction of insects, but also have defense implications for insects [68, 69]. Thus, like common DEGs, many specific DEGs in both *S. avenae* biotypes were shown to have detoxification and defense implications for this aphid on different plants.

Of all the above-mentioned DEGs (common or biotype-specific), we identified many transcripts related to detoxification and defense in aphids, including those encoding for cytochrome P450s (2 in biotype 1 and 8 in biotype 3), carboxylesterase (0 in biotype 1 and 2 in biotype 3), UDP-glucuronosyltransferases (2 in biotype 2 and 6 in biotype 3, 1 co-existed in two biotypes), protease inhibitor, peroxidase, heat shock protein, and etc. (Table 2). This makes sense since different secondary metabolites and toxins in variable plants can induce aphids to differentially express different proteins for detoxification and defense [63, 70–73]. Thus, it seemed that defense-related genes underwent the most intensive expression restructuring for *S. avenae* in response to host plant transfer, suggesting that these genes are the most important candidates for further functional research on the understanding of biotype differentiation in this aphid. For this purpose, correlational analyses between expression of these genes and fitness parameters of *S. avenae* were conducted. Indeed, we found that the fecundity of biotype 1 of this aphid on its alternative plant (i.e., barley) was significantly correlated with gene expression of a cuticle protein (*CP5*) and a cytochrome P450 (*CYP6DA2*). Similarly, the expression of a defense related gene (i.e., *UGT2B2*) was significantly associated with the fecundity of biotype 3 on its alternative plant (i.e., wheat). These results suggested that these defense-related genes were closely related to adaptive potential of *S. avenae* on alternative plants, and thus might play critical roles in the development of various biotypes in this aphid. Interestingly, the enriched GO-terms of DEGs between two biotypes also demonstrated that there has been expression divergence in genes associated with defense (e.g., proteolysis, chitin metabolic process, oxidation-reduction process, and detoxification) for different *S. avenae* biotypes.

In this study, differential expression of defense-related transcripts between the two biotypes of *S. avenae* was not only reflected in the dramatic difference in the number and categories of DEGs, but also in the amount and pattern of plasticity in expression of these genes. Recent studies have shown that the latter can have significant implications for the divergence of aphid biotypes on different host plants [57, 74, 75], but direct evidence is still rare. Our results in this study suggested that expression plasticity of defense related genes might alter vital life-history traits (e.g., fecundity) of *S. avenae* biotypes on different plants. In addition, compared with biotype 1, biotype 3 showed a reduced plastic scope of specific DEGs in response to host transfer, meaning that biotype 3 had relatively lower gene transcriptional plasticity than biotype 1. This extant pattern of reduced transcriptional plasticity in biotype 3 could be indicative of adaptation via genetic assimilation [76]. Another mutually unexclusive explanation is that this pattern may be attributed to selection on plasticity [77]. So, alternative host plants can have potentially selective effects on phenotypic plasticity and the underlying gene expression plasticity in both biotypes. We did identify selective effects of different host plants on life-history trait plasticity of *S. avenae* in previous studies [74]. Therefore, gene expression plasticity in *S. avenae* might be the primary driving force underlying the changing vital life-history traits (e.g., fecundity) of both biotypes on alternative plants [48]. Ultimately, this could have significant impacts on the adaptive potential and differentiation of *S. avenae* biotypes on different plants.

The mechanisms underlying the divergence of variable biotypes in aphids often remain elusive. In our case, the English grain aphid (*S. avenae*) has recently been found to develop into multiple biotypes on both barley and wheat in China [48]. Genetic differentiation between these biotypes is a reasonable assumption, since significant genetic divergence has been found in different geographic populations or host-associated clones for this aphid [20–23]. However, in our most recent study, little genetic differentiation was detected between *S. avenae* biotypes 1 and 3 used in this study [48], which could not explain the divergence of the two biotypes involved. Another possibility is that different aphid biotypes can be associated with different secondary symbionts [41, 78–80], but *S. avenae* biotypes 1 and 3 showed no differential secondary symbiont infections in our study (data not shown). Phenotypic plasticity, common for different populations of aphids, has been thought to facilitate the divergence and evolution of biotypes in aphids [57, 74, 75, 81], but direct and sound evidence is rare. In this study, the amount and pattern of expression plasticity for defense related genes were showed to have potentially important impact on the adaptive potential and differentiation of *S. avenae* biotypes on different plants. Although further studies are still needed to clarify the specific functions of the identified candidate genes potentially important for aphids’ use of resistant hosts and their biotype divergence on different plants, our results did suggest that transcriptional plasticity could be an important mechanism underlying adaptive variation in the development and evolution of aphid biotypes. Our results can provide insights into the role of gene expression plasticity in the divergence of insect biotypes and adaptive evolution of insect populations.

## Conclusions

We conducted transcriptome profiling analyses for two biotypes of *S. avenae* on both original and alternative plants. In response to host plant shift by the two biotypes, 39 DEGs were shared by both biotypes, whereas 126 and 861 DEGs occurred only in biotypes 1 and 3, respectively. Gene enrichment and correlational analyses showed functional divergence in defensive DEGs for the two biotypes in response to host transfer. Biotype 3 had relatively lower gene transcriptional plasticity than biotype 1. Thus, transcriptional plasticity in defense related genes may play critical roles in the phenotypic evolution and development of aphid biotypes. Our results can provide insights into the role of gene expression plasticity in the biotype development and adaptive evolution of insect populations.

## Methods

### Aphid biotypes

Multiple *S. avenae* biotypes (i.e., biotypes 1–6) were identified based on their unique virulence profiles on different wheat (*Triticum aestivum* L.) / barley (*Hordeum vulgare* L.) varieties in our previous study [48]. Due to their differential fitness on Xiyin No.2 (a barley variety, Jiangsu Dahua Seed Enterprise Co., Ltd., Nanjing, Jiangsu Province, China) and Aikang 58 (a wheat variety, Henan Huafeng Seed Industry Science and Technology Co., Ltd., Zhengzhou, Henan Province, China), biotypes 1 and 3 were selected for use in this study. From April to July of 2016, original clones of biotypes 1 and 3 were collected on wheat and barley, respectively (Table S5). Both biotypes were kept on the plant of origin (i.e., wheat or barley) under the following conditions: temperature 22 ± 1 °C, relative humidity 65 ± 5%, and photoperiod 16:8 (L:D) h. In order to minimize confounding environmental effects, all aphid clones were reared under the above-mentioned common laboratory conditions for at least three generations before the experiment. In our previous study, biotype 3 was showed to have higher fecundity on barley (e.g., Xiyin No.2), whereas biotype 1 had higher fecundity on wheat (e.g., Aikang 58), suggesting biotypes 1 and 3 could cause more damage on wheat and barley, respectively [82].

### RNA extraction and sample preparation for sequencing

Test aphid individuals were kept on the plant of origin (i.e., wheat or barley) under the aforementioned environmental conditions. New-born first instar nymphs of both biotypes (i.e., biotypes 1 and 3) were transferred onto two-leaf stage seedlings of Aikang 58 and Xiyin No.2 planted in 200 ml plastic pots [6 cm in diameter, containing turfy soil mixed with vermiculite and perlite (4:3:1, v/v/v)]. Each plastic pot was well enclosed with a transparent plastic cylinder (6 cm in diameter, 15 cm in height) which had a terylene mesh top for ventilation. After molting into adults and feeding for additional 24 h, 10 un-winged aphid individuals were collected each time and put into a 1.5 ml RNase-free tube. Aphid samples in RNase-free tubes were frozen immediately in liquid nitrogen and stored in a freezer at − 80 °C. Three biological replicates were conducted for each *S. avenae* biotype on each test plant. Total RNA was extracted according to the instructions of the MiniBEST Universal RNA Extraction Kit (Takara Bio Inc., Dalian, China), and the potential genomic DNA contamination of total RNA was eliminated with RNase-free DNase I (Takara Bio Inc., Dalian, China). RNA quantity and quality were assessed by using a NanoPhotometer® spectrophotometer (IMPLEN, CA, US) and Bioanalyzer 2100 instrument (Agilent Technologies, CA, US) according to the manufacturers’ instructions.

The cDNA library for each sample was established by using the NEBNext® UltraTM RNA Library Prep Kit for Illumina (NEB, Beverly, MA, US), and the high quality of all cDNA libraries was confirmed with the Agilent Bioanalyzer 2100 system (Agilent Technologies, CA, US). All cDNA libraries were analyzed with the paired-end DNA sequencing technique by using an Illumina HiSeq 2500 system (Illumina, Inc., San Diego, CA) of Ovidson Gene Technology Co., Ltd., Beijing, China. The raw datasets were submitted to the NCBI Sequence Read Archive (SRA) database (SRA accession: PRJNA575173).

### Transcriptome assembly and refinement

Clean reads were obtained by filtering out the adapter sequences, low-quality reads (more than 50% of nucleotides with Qphred ≤20), and those with ambiguous “N” nucleotides > 10%. Reads from all samples were pooled, and the de novo assembly was performed by using TRINITY (v2.1.1) with default parameters as described in [83]. In order to reduce the redundancy, transcripts with 95% similarity were processed by using the software CD-HIT (v4.6.7) [84]. For homology search and annotation, all unigenes were used in search of the following databases: NR (NCBI non-redundant proteins, e-value ≤1.0e-5), NT (NCBI non-redundant nucleotides, e-value ≤1.0e-5), Pfam (protein families, e-value ≤0.01), KOG (eukaryotic orthologous groups, e-value ≤0.001) and Swiss-Prot (e-value ≤1.0e-5). Gene ontology (GO) terms were further analyzed in Blast2GO with a threshold e-value of ≤1.0e-5 [85]. In order to evaluate the completeness of the assembly, the BUSCO (v4.0.2) pipeline was performed against the dataset of conserved genes in insects (i.e., insecta_odb10) [86].

### Analyses of transcriptional plasticity at the transcriptome level

Clean reads from all samples were aligned with Bowtie2 [87], and the expression levels of transcripts were determined by using RSEM (RNA-Seq by Expectation-Maximization) v 1.2.3 [88]. The DESeq2 R package (v1.10.1) was used to model the raw count data with a negative binomial model, and test for differential expression of genes [89]. In this package, the Benjamini-Hochberg method was implemented to calculate adjusted *P*-values (FDR, false discovery rate) [90], and we considered an adjusted *P***-**value less than 0.05 significant. Based on normalized counts, the reproducibility among biological replicates was also assessed by using the plotPCA() function in DESeq2 [89]. Both common (co-existed in the two aphid biotypes) and specific (present in one biotype only) DEGs (differentially expressed unigenes) were analyzed. DEGs between the two biotypes on their original plant (i.e., wheat or barley) were also analyzed, and GO term enrichment analysis for these DEGs were analyzed by using the online tools of GO analysis (http://www.omicshare.com/). Briefly, we used the GO annotation data for the assembly as reference in the enrichment analysis of ‘biological process’ Go terms for sets of DEGs identified above. In this analysis, *P*-values were obtained with the hypergeometric test [91], and adjusted by using the Benjamini-Hochberg correction (FDR < 0.05). To assess the difference between *S. avenae* biotypes for the magnitude of transcriptional changes in response to host plant transfer, we compared the density distributions of Log2 fold changes for both upregulated and downregulated DEGs of the two biotypes. This analysis was implemented by using the “edgeR” package v3.8.6 [92] in R v.3.5.1 [93]. We then tested for significant differences between the two biotypes in the magnitude of Log_2_ fold changes of DEGs by using the nonparametric Wilcoxon rank-sum test in the software SPSS Statistics v.23, providing an estimate of variability in transcriptional plasticity in both biotypes.

### MMC (modulated modularity clustering) analysis

Specific DEGs of each biotype in response to host plant transfer were further analyzed to identify transcriptional modules by using the modulated modularity clustering (MMC) analysis. This analysis can detect modules of putatively co-regulated genes that exhibit correlated transcriptional patterns, and provide insights into the mechanistic underpinnings of complex traits [94, 95]. This analysis was conducted with the MMC package in Python 2.7. The raw count data for above-mentioned DEGs were used as input, and MMC could seek and define the optimal clustering based on a single objective function. Once the modules of co-expressed genes were defined, enriched GO terms associated with each module were analyzed and visualized by using the aforementioned method. This functional enrichment analysis was conducted for all GO categories represented by a minimum of five annotated genes.

### Quantitative real-time PCR (qRT-PCR)

The expression of selected unigenes were also examined by using qRT-PCR. RNA extraction and cDNA synthesis for each sample were conducted following the above-mentioned method. The gene NADH was chosen as the reference gene because of its consistent expression in all samples in this study and in our previous studies [96]. Specific primers for each gene were designed by using Beacon Designer version 8.0 (Premier Biosoft, Palo Alto, CA) (Table S6). As described in [96], 20 μL volume reactions contained 10 μL SYBR Premix Ex Taq II (TaKaRa), 2 μL cDNA, 1 μL each forward and reverse primer (10 μM), and 6 μL ddH2O. All qRT-PCR reactions were performed on a Roche LightCycler® 480 II system (Roche Diagnostics Ltd., Rotkreuz, Switzerland). qRT-PCR cycling conditions were as follows: one cycle of 95 °C for 30 s, and 40 cycles of 95 °C for 5 s followed by 60 °C for 30 s. Melt curve analyses were conducted to confirm the homogeneity of the PCR products. There were three biological and two technical replications for each unigene. The relative expression of selected genes was determined by using the 2^-ΔΔCt^ method [97]. In order for validation of the RNA-Seq data, relative expression levels of unigenes were compared with Student’s *t*–tests, and the relationship between data from RNA-Seq and qRT-RCR was assessed by using the Pearson correlation analysis in the software SPSS Statistics 23.

### Correlations between gene expression and aphid fecundity

New-born first instar nymphs of each biotype were transferred onto single plant seedlings of two-leaf stage under the following conditions: temperature 22 ± 1 °C, relative humidity 65 ± 5%, and photoperiod 16:8 (L:D) h. Test individuals were checked twice daily for molting or reproductive events until day 5 after each test individual initiated the reproduction. Five-day fecundities of test aphid individuals were recorded. At the same time, ten aphid individuals under each treatment were collected in a 1.5 ml RNase-free tube for gene expression analysis. RNase-free tubes with collected aphids were frozen immediately in liquid nitrogen and stored in a freezer at − 80 °C. Twelve replicates were conducted for each *S. avenae* biotype on each test plant (i.e., wheat or barley). RNA extraction and cDNA synthesis for each sample were conducted following the above-mentioned method. The expression of target genes were examined by using qRT-PCR mentioned above. Pearson correlations between five-day fecundity and gene expression were assessed with the software SPSS Statistics 23.

## Supplementary information

**Additional file 1 **: **Table S1.** Statistics of RNA-Seq for *Sitobion avenae* biotypes 1 and 3 feeding on wheat and barley; **Table S2.** The Log2 fold changes and adjusted *P* values of specific DEGs related to defense in biotype 1 responding to host transfer; **Table S3.** The Log2 fold changes and adjusted *P* values of specific DEGs related to defense in biotype 3 responding to host transfer; **Table S4.** Gene enrichment analysis of biological processes GO-terms for transcriptional modules of specific DEGs in both biotypes of *Sitobion avenae*; **Table S5.** Sample collection information for *Sitobion avenae* biotypes 1 and 3; **Table S6.** Primer sequences for selected genes in qRT-PCR; **Figure S1.** BUSCO analysis for the Trinity assembly; **Figure S2.** Assessment of reproducibility among biological replicates; **Figure S3.** Validation of RNA-Seq analyses with qRT-PCR.

## Data Availability

The raw datasets were submitted to the NCBI Sequence Read Archive (SRA) database (SRA accession: PRJNA575173).

## Abbreviations

DEGs: Differentially expressed unigenes
FDR: False discovery rate
GO: Gene ontology
NCBI: National center for biotechnology information
MMC: Modulated modularity clustering
qRT-PCR: Quantitative reverse transcription-polymerase chain reaction
AW: Biotype 1 on wheat
AB: Biotype 1 on barley
BW: Biotype 3 feeds on wheat
BB: Biotype 3 feeds on barley
UGT: UDP-glucuronosyltransferase
FMO: Flavin-containing monooxygenase gene
GST: Glutathione S-transferase
ALP: Alkaline phosphatase gene
CBCP: Cathepsin B-like cysteine proteinase
TPS: Alpha-trehalose-phosphate synthase
CYB5: Cytochrome b5
Zrt ZIP1: Zinc transporter ZIP1
CP: Cuticle protein; CYP Cytochrome P450
ABCG: ABC transporter G family member
NADH: Nicotinamide adenine dinucleotide
