# Supplementary material for: Transcriptome profiling revealed potentially important roles of defensive gene expression in the divergence of insect biotypes: a case study with the cereal aphid Sitobion avenae
Source: BMC Genomics. 2020 Aug 6;21:546. doi: 10.1186/s12864-020-06950-y (PMC7430832; doi:10.1186/s12864-020-06950-y)
Supplement: Supplementary file 1 — Additional file 1 : Table S1. Statistics of RNA-Seq for Sitobion avenae biotypes 1 and 3 feeding on wheat and barley; Table S2. The Log2 fold changes and adjusted P values of specific DEGs related to defense in biotype 1 responding to host transfer; Table S3. The Log2 fold changes and adjusted P values of specific DEGs related to defense in biotype 3 responding to host transfer; Table S4. Gene enrichment analysis of biological processes GO-terms for transcriptional modules of specific DEGs in both biotypes of Sitobion avenae; Table S5. Sample collection information for Sitobion avenae biotypes 1 and 3; Table S6. Primer sequences for selected genes in qRT-PCR; Figure S1. BUSCO analysis for the Trinity assembly; Figure S2. Assessment of reproducibility among biological replicates; Figure S3. Validation of RNA-Seq analyses with qRT-PCR. [file 12864_2020_6950_MOESM1_ESM.docx]

**Table S1** Statistics of RNA-Seq for *Sitobion avenae* biotypes 1 and 3 feeding on wheat and barley

| **Biotype** | **Plant** | **Sample**  **ID** | **Raw**  **reads** | **Clean**  **reads** | **Error**  **(%)** | **Q20**  **(%)** | **Q30**  **(%)** | **GC**  **(%)** | **Mapped reads (%)** |
| --- | --- | --- | --- | --- | --- | --- | --- | --- | --- |
| 1 | Wheat | AW1 | 28117345 | 27506078 | 0.01 | 98.80 | 96.58 | 40.99 | 72.29 |
| 1 | Wheat | AW2 | 26112244 | 25626848 | 0.01 | 98.77 | 96.53 | 40.04 | 71.67 |
| 1 | Wheat | AW3 | 25989771 | 25482373 | 0.01 | 98.76 | 96.49 | 40.32 | 72.46 |
| 1 | Barley | AB1 | 26108796 | 25669614 | 0.01 | 98.72 | 96.32 | 40.38 | 72.93 |
| 1 | Barley | AB2 | 28572370 | 27993146 | 0.01 | 98.80 | 96.59 | 40.46 | 73.23 |
| 1 | Barley | AB3 | 29031747 | 28512017 | 0.01 | 98.79 | 96.57 | 40.46 | 72.73 |
| 3 | Wheat | BW1 | 26056761 | 25567593 | 0.01 | 98.77 | 96.54 | 40.19 | 72.80 |
| 3 | Wheat | BW2 | 28490615 | 27982220 | 0.01 | 98.79 | 96.58 | 40.24 | 72.80 |
| 3 | Wheat | BW3 | 28040119 | 27432672 | 0.01 | 98.66 | 96.18 | 40.34 | 72.84 |
| 3 | Barley | BB1 | 28413038 | 27900884 | 0.01 | 98.78 | 96.56 | 39.78 | 72.22 |
| 3 | Barley | BB2 | 28730442 | 28094826 | 0.01 | 98.73 | 96.47 | 39.70 | 72.49 |
| 3 | Barley | BB3 | 40153204 | 39420648 | 0.01 | 98.70 | 96.40 | 39.96 | 73.14 |
| Total number of unigenes | | | | | 143058 | | | | |
| Total length of unigenes (nt) | | | | | 95512520 | | | | |
| Mean length of unigenes (nt) | | | | | 358 | | | | |
| N50 of unigenes | | | | | 1012 | | | | |

Note: Q20 and Q30, percentage of bases with quality over a Phred score of 20 (i.e., an error rate of 1%) and 30 (i.e., an error rate of 0.1%), respectively; GC, proportion of G and C bases; N50, length N for which 50% of all bases in the assembly are located in a transcript of length L < N.

**Table S2** The Log2 fold changes and adjusted *P* values of specific DEGs related to defense in biotype 1 responding to host transfer

| **Gene ID** | **Annotation** | **Log_2_ (Fold**  **change) ^a^** | **Adjusted *P* value** |
| --- | --- | --- | --- |
| c92410_g2 | Cytochrome P450 6k1-like | -0.80 | < 0.001 |
| c77510_g1 | Cytochrome P450 6DA2 | -0.42 | 0.004 |
| c94527_g1 | UDP-glucuronosyltransferase 2B17 | -0.36 | 0.024 |
| c85222_g1 | Laccase-5 | 0.18 | 0.027 |
| c89079_g2 | Zinc transporter ZIP1 | 0.69 | 0.001 |
| c90124_g1 | Heat shock 70 kda protein | 0.25 | < 0.001 |
| c77784_g2 | Cuticle protein 27-like | 0.40 | 0.043 |
| c63100_g1 | Cuticle protein 5-like | 0.60 | 0.011 |
| c80892_g1 | Venom serine protease | 0.37 | 0.013 |
| c90342_g4 | Cathepsin B-like cysteine proteinase 4 | 1.43 | 0.001 |

^a^ Fold changes of gene expression in biotype 1 on barley compared with wheat.

**Table S3.** The Log2 fold changes and adjusted *P* values of specific DEGs related to defense in biotype 3 responding to host transfer

| **Gene ID** | **Annotation** | **Log_2_ (Fold change) ^a^** | **Adjusted *P* value** |  | **Gene ID** | **Annotation** | **Log_2_ (Fold change) ^a^** | **Adjusted *P* value** |
| --- | --- | --- | --- | --- | --- | --- | --- | --- |
| c94592_g2 | Cytochrome P450 4C1-like | -0.92 | < 0.001 |  | c68074_g1 | Superoxide dismutase | -0.24 | 0.001 |
| c90459_g1 | Cytochrome P450 4C1-like | -0.73 | < 0.001 |  | c88468_g2 | Laccase-5 | -1.33 | 0.038 |
| c84626_g1 | Cytochrome P450 CYP6CY3 | -0.65 | 0.046 |  | c83036_g1 | Laccase-4 | -0.40 | 0.004 |
| c85444_g1 | Cytochrome P450 4g15 | -0.54 | < 0.001 |  | c93734_g3 | Serine protease inhibitor | 0.14 | 0.015 |
| c89199_g1 | Cytochrome P450 6B1-like | -0.49 | < 0.001 |  | c100733_g1 | Cutile protein | -0.58 | < 0.001 |
| c94331_g1 | Cytochrome P450 6a14 | -0.24 | 0.030 |  | c85485_g2 | Cuticle protein 19-like | -0.56 | < 0.001 |
| c80471_g1 | Cytochrome P450 49a1 | 0.16 | 0.010 |  | c92557_g3 | Cuticle protein H1C-like | -0.39 | 0.036 |
| c82766_g1 | Cytochrome P450 6DA1 | 0.34 | < 0.001 |  | c91421_g3 | Cuticle protein 18.6-like | -0.22 | 0.004 |
| c92686_g1 | Esterase FE4-like | -0.71 | < 0.001 |  | c77784_g2 | Cuticle protein 27-like | -0.04 | 0.952 |
| c89160_g1 | Esterase FE4-like | -0.62 | < 0.001 |  | c76629_g1 | Cuticle protein 5 | -0.35 | 0.027 |
| c88302_g1 | Esterase E4 | -0.49 | < 0.001 |  | c91421_g4 | Cuticle protein 7-like | -0.25 | 0.024 |
| c94328_g3 | Esterase E4-like | -0.45 | 0.046 |  | c94379_g1 | Cuticle protein | -0.22 | 0.044 |
| c82415_g1 | Venom carboxylesterase-6 | -0.29 | 0.005 |  | c87221_g1 | Cuticle protein 16.5-like | -0.22 | 0.018 |
| c85450_g1 | Venom carboxylesterase-6-like | -0.26 | 0.007 |  | c73836_g2 | Cuticular protein | -0.96 | 0.028 |
| c83802_g1 | Glutathione S-transferase 1-1-like | -0.35 | < 0.001 |  | c92557_g1 | Cuticular protein | -0.67 | 0.037 |
| c89914_g1 | UDP-glucuronosyltransferase 2C1-like | -0.52 | 0.029 |  | c5073_g1 | Cuticular protein CPG12-like | -0.65 | 0.006 |
| c91825_g1 | UDP-glucuronosyltransferase 2B7-like | -0.50 | 0.003 |  | c84129_g1 | Cuticular protein RR-1 | -0.39 | 0.001 |
| c92512_g4 | UDP-glucuronosyltransferase 2B2 | -0.37 | 0.021 |  | c88076_g3 | Cuticular protein 47 | -0.30 | 0.041 |
| c82277_g1 | UDP-glucuronosyltransferase 2B1 | -0.25 | < 0.001 |  | c85676_g1 | Zinc transporter ZIP1 isoform X2 | -0.22 | 0.007 |
| c90721_g1 | UDP-glucuronosyltransferase 2B33 | 1.09 | 0.030 |  | c85468_g1 | Zinc transporter 1 | 0.31 | 0.002 |
| c76175_g1 | ABC transporter G family member 23 | -0.77 | < 0.001 |  | c86349_g2 | Heat shock protein | -0.27 | 0.025 |
| c93673_g2 | ABC transporter G family member 20-like | -0.43 | 0.001 |  | c61865_g1 | Heat shock protein | -0.27 | 0.032 |
| c94097_g2 | ABC transporter G family member 20 | -0.24 | < 0.001 |  | c86175_g2 | Heat shock protein beta-1 | -0.25 | < 0.001 |
| c85139_g1 | Cytochrome b5 reductase 2 | -0.21 | 0.003 |  | c91279_g1 | Heat shock protein | -0.22 | < 0.001 |
| c91846_g13 | Peroxidase-like | -0.92 | 0.030 |  | c58997_g1 | Heat shock protein | -0.20 | < 0.001 |
| c84574_g1 | Chorion peroxidase-like | -0.54 | < 0.001 |  | c90905_g1 | Heat shock protein | 0.32 | < 0.001 |
| c83707_g1 | Peroxidase-like | -0.28 | < 0.001 |  | c88798_g1 | Serine protease | -0.57 | 0.028 |
| c87773_g1 | Peroxidase-like | -0.26 | 0.011 |  | c86944_g1 | Serine protease K12H4.7 | -0.41 | < 0.001 |
| c87324_g1 | Peroxidase-like | -0.25 | < 0.001 |  | c94161_g5 | Alpha,alpha-trehalose-phosphate synthase | -0.35 | < 0.001 |

^a^ Fold changes of gene expression in biotype 3 on wheat compared with barley

**Table S4.** Gene enrichment analysis of biological processes GO-terms for transcriptional modules of specific DEGs in both biotypes of *Sitobion avenae*

| **Module** | **GO** | **Go_description** | **DE** | **Total** | ***P*-value** | **FDR** |
| --- | --- | --- | --- | --- | --- | --- |
| Specific plastic genes of biotype 1 | | | | | | |
| P1 | GO:0016999 | Antibiotic metabolic process | 1 | 5 | 0.002 | 0.019 |
| P1 | GO:0017001 | Antibiotic catabolic process | 1 | 5 | 0.002 | 0.019 |
| P1 | GO:0017144 | Drug metabolic process | 1 | 5 | 0.002 | 0.019 |
| P1 | GO:0007269 | Neurotransmitter secretion | 1 | 6 | 0.003 | 0.019 |
| P1 | GO:0023061 | Signal release | 1 | 6 | 0.003 | 0.019 |
| P1 | GO:0099531 | Presynaptic process involved in synaptic transmission | 1 | 6 | 0.003 | 0.019 |
| P1 | GO:0001505 | Regulation of neurotransmitter levels | 1 | 8 | 0.004 | 0.023 |
| P1 | GO:0007268 | Synaptic transmission | 1 | 11 | 0.005 | 0.025 |
| P1 | GO:0099536 | Synaptic signaling | 1 | 11 | 0.005 | 0.025 |
| P1 | GO:0099537 | Trans-synaptic signaling | 1 | 11 | 0.005 | 0.025 |
| P1 | GO:0007267 | Cell-cell signaling | 1 | 16 | 0.007 | 0.035 |
| P1 | GO:0006836 | Neurotransmitter transport | 1 | 23 | 0.010 | 0.047 |
| P2 | GO:0006508 | Proteolysis | 6 | 944 | < 0.001 | 0.001 |
| Specific plastic genes of biotype 3 | | | | | | |
| T1 | GO:0006457 | Protein folding | 7 | 219 | < 0.001 | < 0.001 |
| T2 | GO:0006414 | Translational elongation | 5 | 120 | < 0.001 | 0.017 |
| T2 | GO:0006412 | Translation | 12 | 1188 | < 0.001 | 0.049 |
| T2 | GO:0043043 | Peptide biosynthetic process | 12 | 1198 | 0.001 | 0.049 |
| T2 | GO:0043604 | Amide biosynthetic process | 12 | 1244 | 0.001 | 0.049 |
| T2 | GO:0006518 | Peptide metabolic process | 12 | 1246 | 0.001 | 0.049 |
| T4 | GO:0006030 | Chitin metabolic process | 6 | 82 | < 0.001 | 0.003 |
| T4 | GO:1901071 | Glucosamine-containing compound metabolic process | 6 | 92 | < 0.001 | 0.003 |
| T4 | GO:0006022 | Aminoglycan metabolic process | 6 | 100 | < 0.001 | 0.003 |
| T4 | GO:0006040 | Amino sugar metabolic process | 6 | 101 | < 0.001 | 0.003 |
| T4 | GO:0051246 | Regulation of protein metabolic process | 7 | 205 | < 0.001 | 0.016 |
| T4 | GO:0001731 | Formation of translation preinitiation complex | 3 | 25 | < 0.001 | 0.030 |
| T4 | GO:0006446 | Regulation of translational initiation | 3 | 26 | < 0.001 | 0.030 |
| T4 | GO:0032268 | Regulation of cellular protein metabolic process | 6 | 193 | 0.001 | 0.049 |
| T7 | GO:0055114 | Oxidation-reduction process | 17 | 1680 | < 0.001 | 0.005 |
| T8 | GO:0044765 | Single-organism transport | 14 | 1741 | < 0.001 | 0.032 |
| T8 | GO:1902578 | Single-organism localization | 14 | 1759 | < 0.001 | 0.032 |
| T8 | GO:0055085 | Transmembrane transport | 11 | 1201 | < 0.001 | 0.040 |
| T9 | GO:0006810 | Transport | 39 | 2457 | < 0.001 | 0.008 |
| T9 | GO:0051234 | Establishment of localization | 39 | 2463 | < 0.001 | 0.008 |
| T9 | GO:0051179 | Localization | 39 | 2497 | < 0.001 | 0.008 |
| T9 | GO:0055085 | Transmembrane transport | 24 | 1201 | < 0.001 | 0.008 |

Note: Modules P3, P4, P5, T3, T5 and T6 were not enriched for any terms.

**Table S5.** Sample collection information for *Sitobion avenae* biotypes 1 and 3

| **Biotypes** | **Sample Size** | **Host** | **Sampling location** | **GPS Coordinates** | **Collection Date** |
| --- | --- | --- | --- | --- | --- |
| **Biotypes 1** | 5 | Wheat | Ganzhou District, Zhangye City, Gansu Province | 100°28′ E; 38°54′ N | 2016.04 |
| **Biotypes 1** | 3 | Wheat | Minle County, Zhangye City, Gansu Province | 100°46′ E; 38°25′ N | 2016.04 |
| **Biotypes 1** | 4 | Wheat | Gaotai County, Zhangye City, Gansu Province | 99°41′ E; 39°25′ N | 2016.07 |
| **Biotypes 3** | 3 | Barley | Biyang County, Zhumadian City, Henan Province | 113°20′ E; 32°46′ N | 2016.04 |
| **Biotypes 3** | 4 | Barley | Yicheng District, Zhumadian City, Henan Province | 114°02′ E; 33°00′ N | 2016.04 |
| **Biotypes 3** | 3 | Barley | Xincai County, Zhumadian City, Henan Province | 115°03′ E; 32°45′ N | 2016.04 |

**Table S6** Primer sequences for selected genes in qRT-PCR

| **Genes** | **Forward primer (5’ → 3’)** | **Reverse primer (5’ → 3’)** |
| --- | --- | --- |
| ***CP5*** | GGTGTAATCAGAGTATGCGTAT | GCCGAAGAAGGAGTAAGAG |
| ***CBCP4-4*** | TGCCGCTTACGACAACTTA | CCAACACGATCCACAATTCC |
| ***CYP6DA2*** | CGCCATCTATTCGCTGTA | ATCGGAAGTCCTTTACCAT |
| ***CYP6K1*** | GCAATTCTCTTGTTCGCCGCTGGTA | CATCGAGCATATCTCCGCACGCATT |
| ***Zrt ZIP1*** | CGGACAGGACGAAGCCATT | TAGAGTGCCAACGCCAATACC |
| ***CYB5R2*** | TTCCATTGCCACTTATTC | AACATCATTGTCACTACTG |
| ***CYP49A1*** | AGGAGGAGGTGGTGGATA | CGTTGTCTTGGTGAGTAGC |
| ***CYP6DA1*** | ACCAACATATTCGCTACAT | GAATCGCAGACCTATACAA |
| ***ABCG20*** | AATCTCAGTGGTGGTCAACAA | ACGGAGTAACGGGTCTACA |
| ***CYP6A14*** | AGTGTTCGTGTTCTTCCT | ATTGGCTGTCTCGTTGAT |
| ***UGT2B33*** | CTCGCACAATCCGCAAGA | GCATAGCACATACATCCTGGTT |
| ***Esterase E4-1*** | ATCAAGTAGCCGCATTACGTTGGGT | AGCACCTCCTGAACTTCCACCAGAA |
| ***UGT2B2*** | AATCCACTGAAGGAAGCATTAGC | CCTGATATACCTCCGTGACTGAT |
| ***Esterase E4-2*** | TGGAGGAGTCAGTGTTGGCTTAC | AGACAGTGATGCGGCTCTACG |
| ***NADH*** | CGAGGAGAACATGCTCTTAGAC | GATAGCTTGGGCTGGACATATAG |


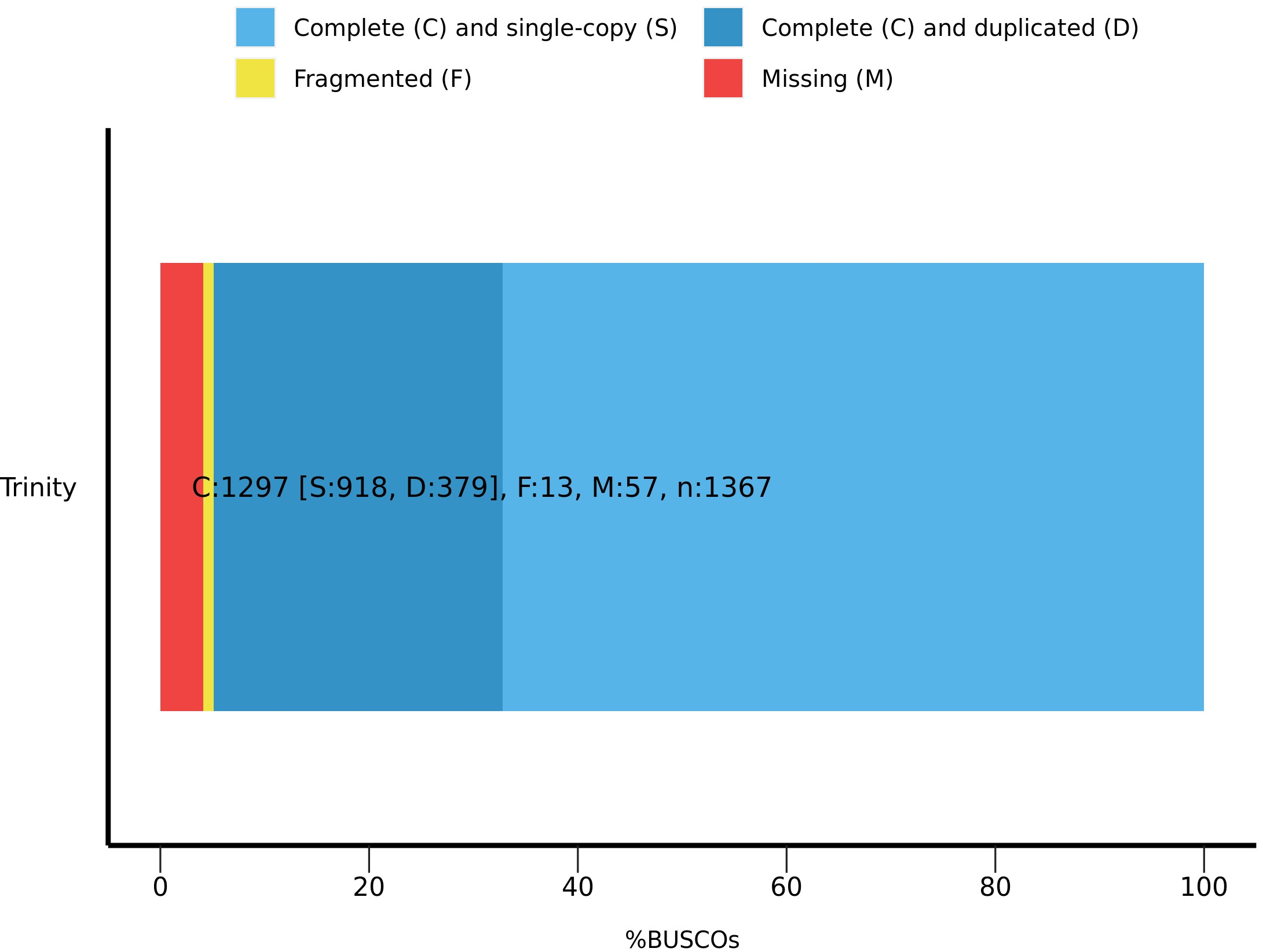


**Fig. S1.** BUSCO analysis for the Trinity assembly


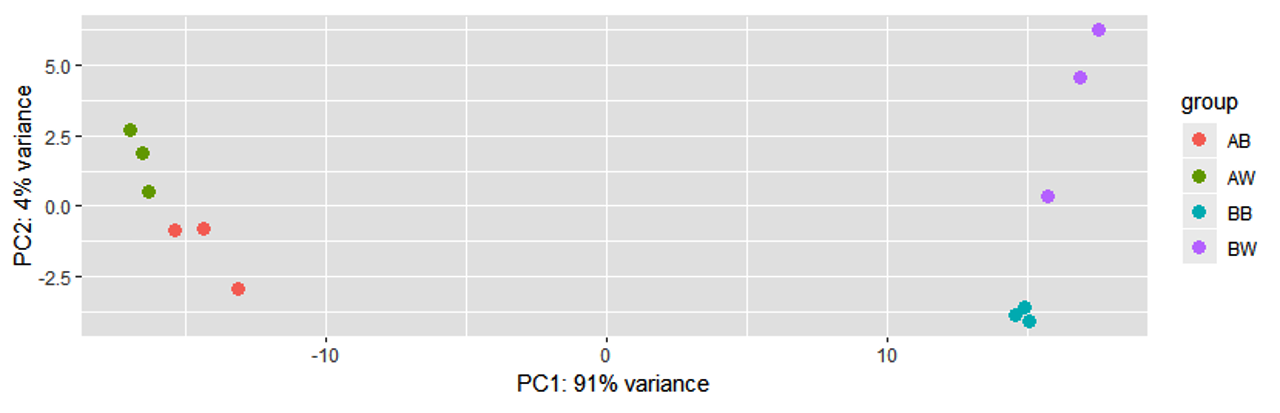


**Fig. S2.** Assessment of reproducibility among biological replicates (AB, biotypes 1 feed on barley; AW, biotypes 1 feed on wheat; BB, biotypes 3 feed on barley; BW, biotypes 3 feed on wheat)


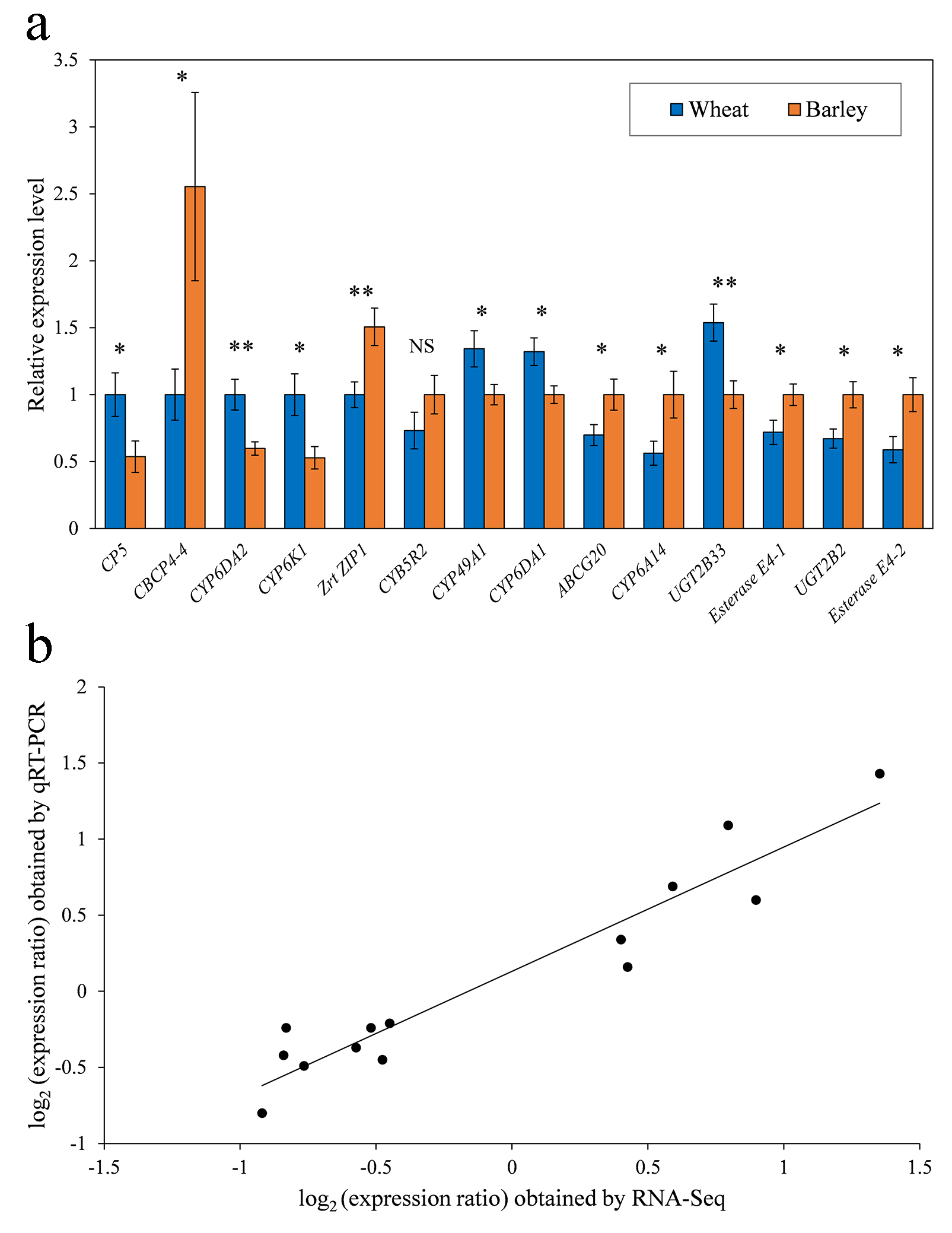


**Fig. S3.** Validation of RNA-Seq analyses with qRT-PCR (a, comparisons between gene expression levels before and after host transfer by using qRT-PCR; b, Pearson’s correlation between fold changes from both qRT-PCR and RNA-Seq analyses [For the upper panel, the first five genes were selected from biotype 1-specific DEGs, and the other nine genes were selected from biotype 3-specific DEGs (one from each transcriptional module); *CP5*, cuticle protein 5-like, c63100_g1; *CBCP4-4*, cathepsin B-like cysteine proteinase 4, c90342_g4; *CYP6DA2*, cytochrome P450 6DA2, c77510_g1; *CYP6K1*, cytochrome P450 6k1-like, c92410_g2; *Zrt ZIP1*, zinc transporter ZIP1, c89079_g2; *CYB5R2*, cytochrome b5 reductase 2, c85139_g1; *CYP49A1*, cytochrome P450 49a1, c80471_g1; *CYP6DA1*, cytochrome P450 6DA1, c82766_g1; *ABCG20*, ABC transporter G family member 20, c94097_g2; *CYP6A14*, cytochrome P450 6a14, c94331_g1; *UGT2B33*, UDP-glucuronosyltransferase 2B33, c90721_g1; *Esterase E4-1*, Esterase E4-like, c94328_g3; *UGT2B2*, UDP-glucuronosyltransferase 2B2, c92512_g4; *Esterase E4-2*, Esterase E4,c88302_g1; *, *P* < 0.05; **, *P* < 0.01; ***, *P* < 0.001; NS, non-significant]
